# Effect of Fiber Cross-Sectional and Surface Properties on the Degradation of Biobased Polymers

**DOI:** 10.3390/polym16213096

**Published:** 2024-11-02

**Authors:** Simon Schick, Andreas Weinberger, Robert Groten, Gunnar H. Seide

**Affiliations:** 1 Aachen-Maastricht Institute for Biobased Materials (AMIBM), Faculty of Science and Engineering, Maastricht University, Brightlands Chemelot Campus, Urmonderbaan 22, 6167RD Geleen, The Netherlands; s.schick@maastrichtuniversity.nl; 2 International Fibers Group (IFG) ASOTA, Schachermayerstr 22, 4070 Linz, Austria; 3 Department of Textile and Clothing Technology, Niederrhein University of Applied Sciences, Campus Mönchengladbach, Webschulstrasse 31, 41065 Mönchengladbach, Germany

**Keywords:** biopolymer, biodegradable fiber, melt spinning, polylactic acid, polybutylene succinate, cross-section, multi-step degradation

## Abstract

Biobased polymers such as polylactic acid (PLA) and polybutylene succinate (PBS) break down naturally under certain environmental conditions. The efficiency of degradation can be linked directly to fiber surface properties, which influence polymer accessibility. Here, the degradation of PLA and PBS fibers with six different cross-sections was investigated. The fibers were aged by hydrolysis and UV exposure in an accelerated weathering test, followed by an ISO 20200 laboratory-scale disintegration test with non-aged fibers as controls. The polymers were analyzed by differential scanning calorimetry, Fourier transform infrared spectroscopy, and gel permeation chromatography, comparing the polymer granulate, virgin fibers, and UV-exposed fibers. It was found that the molecular mass and crystallinity of PBS changed more than PLA during spinning. Several PLA samples were completely degraded, whereas all the PBS samples remained intact. Furthermore, surface openings appeared on the PLA fibers during weathering, suggesting greater sensitivity to UV exposure and hydrolysis than PBS. A clear correlation between the fiber surface area and the degradation rate was observed for all samples, but the correlation was positive for PLA and negative for PBS. The slower degradation of PBS fibers with a larger surface area may reflect the ability of PBS to preserve itself by further crystallization during degradation processes at temperatures higher than the glass transition point. The data clearly show that the analysis of single degradation mechanisms is insufficient to predict the behavior of material under real-world conditions, where different degradation mechanisms may work in parallel or consecutively, and may show interdependencies.

## 1. Introduction

The global production of plastics increased by 640% between 1975 and 2019, reaching 338 million tons [1]. An estimated 80% of all plastics produced between 1950 and 2015 have ended up in the environment, resulting in the accumulation of plastic waste [2]. The environmental impact of plastics could be reduced by using biopolymers [3,4]. These are produced from renewable raw materials and some of them also break down naturally under certain environmental conditions. With a 20.7% market share, polylactic acid (PLA) is the most widely used biopolymer suitable for industrial-scale melt electrospinning, and it can also be composted under industrial conditions [5,6]. In contrast, polybutylene succinate (PBS) is the best-performing home-compostable biopolymer, making it a suitable alternative to fossil-based polymers for home applications [7,8].

Polymer degradation involves chemical and physical cleavage of the backbone to form smaller chains with a lower molecular weight. The susceptibility of a polymer to degradation is determined by its structure, and knowledge of the degradation process can be useful for polymer recycling or conversion to valuable hydrocarbon derivatives, preventing environmental pollution [9,10,11]. Polymer degradation often involves a combination of thermal activation, enzyme activity, hydrolysis, oxidation, photolysis, and radiolysis [12]. PLA and PBS break down naturally because their ester groups make them particularly susceptible to hydrolysis [13].

The degradation of polymer fibers usually begins at the surface, because the surface is exposed to microbes and their enzymes [14,15] as well as physical mechanisms such as UV-based photolysis and hydrolysis [16,17]. Accordingly, there is a clear correlation between a fiber’s surface area, mass, and degradation rate. The degradation of fibers in nature rarely involves only a single mechanism. However, the primary mechanisms are usually UV photolysis and hydrolysis caused by exposure to rain, followed by the activity of microbes and their enzymes in the soil. The testing of polymer products for degradability usually considers only a single mechanism in isolation and rarely compares virgin samples to those pre-loaded by exposure to UV or other factors.

The degradation of PLA has been widely studied under industrial composting conditions such as 58 ± 2 °C and 50–55% humidity [18] based on DIN EN ISO 14855-1 [19]. Previous studies have considered pure PLA, PLA/PBS blends, and blends of PLA with polyhydroxyalkanoate (PHA) in an industrial composting test and a burial test in topsoil at 23 °C for 12 weeks [18]. Various forms of PLA have been shown to break down efficiently during industrial composting, including 81 ± 10% of 10 × 10 mm bottle flakes within 58 days [20], 86% of 20 × 20 × 0.3 mm particles within 120 days [21], and 75% of 2 mm film pieces (50 mg each) within 90 days [22]. Similarly, 90% of PBS particles degraded within 160 days [23] when tested according to ASTM 6400 [24]. A comparison of 50 µm PBS film pieces and 200 µm PBS powder alongside 55 µm PLA film pieces and 200 µm PLA powder showed that more of the PBS (18%) than the PLA (12%) was degraded after 45 days [25]. If the temperature of industrial composting is lower, PLA breaks down more slowly [13,26,27]. If the temperature is reduced from 58 to 25 °C, the carbon mineralization rate of PLA is 0.17 mg/day, resulting in 16% degradation within 180 days [28], whereas 60% of PBS breaks down within 180 days at 30 °C [29]. These studies considered films, particles, or powders, and generally found that degradation was accelerated by a larger surface area, but the relationship between surface area and degradation rate is also relevant to fibers [30]. The influence of cross-sectional properties has been considered for partially oriented yarns [31] and in the context of fabric surface characteristics associated with friction–roughness, yarn bending, and rigidity, and the overall structure of the knitted loop [32]. PLA hollow fibers have been examined as drug delivery systems [33]. However, the studies discussed above have not considered the degradation rate as a consequence of fiber cross-sectional properties.

Here, we address this knowledge gap by directly comparing PLA and PBS fibers with the same cross-sectional properties spun on the same machine using identical settings, followed by accelerated weathering and further disintegration tests. We compared virgin fibers to those exposed to accelerated weathering in order to isolate the influence of accelerated weathering on subsequent degradation. This test simulates the improper disposal of a product primarily exposed to environmental factors such as UV photolysis and rain-induced hydrolysis before degradation occurs in the soil. We also compared fibers with different surface areas to confirm the relationship with the degradation rate. This will enable the manufacture of fibers with tailored degradation rates, offering the possibility to design fibers for application-specific degradation under specific environmental conditions. Accordingly, PLA and PBS would make excellent choices for the development of disposable products that do not accumulate in the environment and cause pollution [34].

## 2. Materials and Methods

### 2.1. Materials and Samples

**Figure 1:**
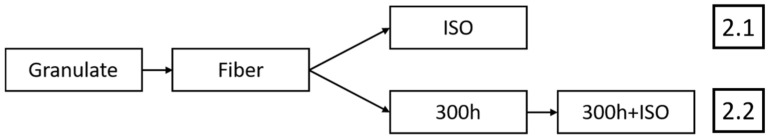
Samples and degradation processes [35]. Pathway 2.1 progresses from granulate to fiber and finally to disintegration (ISO). In contrast, pathway 2.2 includes artificial accelerated weathering (300h) before disintegration (300h+ISO).

PBS FZ91PM and PLA 6202D granulate polymers were provided by IFG ASOTA GmbH Linz Austria. The fibers were allocated codes (Table 1) referring to the polymer (PLA or PBS) followed by an underscore and a description of the fiber cross-section (R1–R4 for circular sections with decreasing diameter, C for C-shaped sections, and T for trilobal sections) followed by another underscore and the degradation pathway (Figure 1). Three samples were used for each measurement.

### 2.2. Differential Scanning Calorimetry (DSC)

DSC was carried out using a Mettler Toledo (Columbus, OH, USA) instrument to determine the melting temperature (*T_m_*) and melt enthalpy (Δ*H_m_*) in three heating–cooling–heating cycles as previously described [35]. The crystallinity (*X_C_*) was calculated using Equation (1) [36], where Δ*H_m_* is the melt enthalpy of the first heating cycle and ${∆H}_{m}^{100}$ is the melt enthalpy of the 100% crystalline polymer (93.7 J/g for PLA and 110.3 J/g for PBS) [37].

(1) $$X_{C}=\;\frac{{∆H}_{m}}{{∆H}_{m}^{100}}*100$$

### 2.3. Fourier Transform Infrared (FTIR) Spectroscopy

The chemical profiles of samples in both pathways were determined using an Alpha 2 FTIR device (Bruker, Billerica, MA, USA) with a single diamond ATR system as previously described [35]. The peak height ratio was calculated using Equation (2) [38].

(2) $$Peak\;height\;ratio=\frac{{Height}_{Peak\;X}}{{Height}_{Reference\;peak}}$$

The carbonyl, hydroxyl, and vinyl indices were calculated using Equations (3), (4), and (5), respectively [38,39,40].

(3) $$Carbonyl\;index\;\left(CI\right)=\;\frac{I_{1710-1713}}{I_{2945}}$$

(4) $$Hydroxyl\;index\;\left(HI\right)=\;\frac{I_{3423-3429}}{I_{2945}}$$

(5) $$Vinyl\;index\;\left(VI\right)=\;\frac{I_{917}}{I_{2945}}$$

### 2.4. Gel Permeation Chromatography (GPC)

The number-average molecular weight (*M_n_*) of each polymer at each processing step was determined by GPC as previously described [35] using a 1260 Infinity System (Agilent Technologies, Santa Clara, CA, USA). The M_n_ enabled us to calculate the degradation parameter *K* using Equation (6) [36].

(6) $$K=\;\frac{M_{n}\;unprocessed}{M_{n}\;processed}$$

### 2.5. Tensile Test

The tensile force and elongation of each sample were determined as previously described [35] according to DIN EN ISO 5079 [41].

### 2.6. Sample Preparation

**Figure 2:**
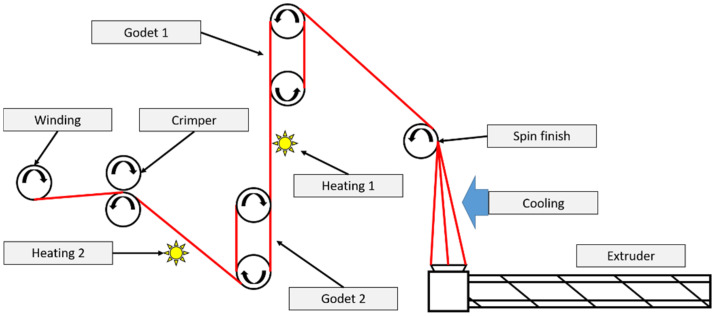
Schematic drawing of the laboratory-scale spinning line.

Our laboratory spinning equipment (Figure 2) was used to prepare all fiber samples by applying the parameters (optimized for process stability and high-quality fibers) listed in Table 1.

**Figure 3:**
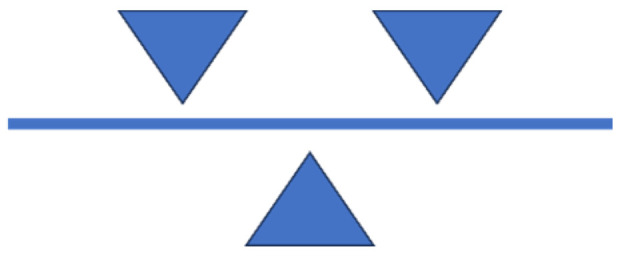
Mechanical bonding pattern (needle entrance direction) [35].

The extrusion temperature was measured before the melt exited the extruder. All fibers were produced with a crimp for further processing. After mechanical testing, the fibers were cut and converted into a nonwoven material, mechanically bonded by needle punching as previously described [35] using the pattern shown in Figure 3, and then cut into 100 cm^2^ samples for FTIR analysis or to match the holder in the UV tester (4.5 × 13.5 cm).

### 2.7. Accelerated Weathering

Accelerated weathering was applied to duplicate samples using a Q-Sun XE-2HS device (Q-Lab, Westlake, OH, USA) as previously described [35]. The overall loading of the samples with UV radiation was similar to the accelerated weathering of plastics under DIN standard EN ISO 11341 [42] (for the light cycle) and ISO 4892-2 [43] (for spraying) or in ISO 4892.3 [44], and was set to simulate an exposure period of several months or years in days [45].

### 2.8. Laboratory-Scale Disintegration

Disintegration tests were carried out as previously described [35] according to DIN standard EN ISO 20200 [46].

### 2.9. Scanning Electron Microscopy (SEM)

Fiber cross-sections were examined using a Jeol (Tokyo, Japan) JSM-IT200 scanning electron microscope. Each fiber was embedded in LX112 resin, cut with a diamond blade on an EM UC7 ultramicrotome (Leica Microsystems, Wetzlar, Germany), and then coated with 4 nm of carbon using a Leica ACE600 sputter coater before imaging. The circumference and cross-sectional area of fibers were determined using ImageJ. The circumference was used to determine the titer (Equation (7) [47]) and the circumference/cross-sectional ratio (CCR) was calculated using Equation (8).

(7) $$Titer\;\left[dtex\right]=r^{2}\left[\mathrm{mm}\right]*\prod *Density\;\left[\frac{g}{cm^{3}}\right]*10,000$$

(8) $$Circumference/cross-sectional\;ratio\;\left(CCR\right)=\frac{Fiber\;circumference}{Fiber\;cross-sectional\;area}$$

## 3. Results

### 3.1. Physical Properties of the Spun Fibers

**Figure 4:**
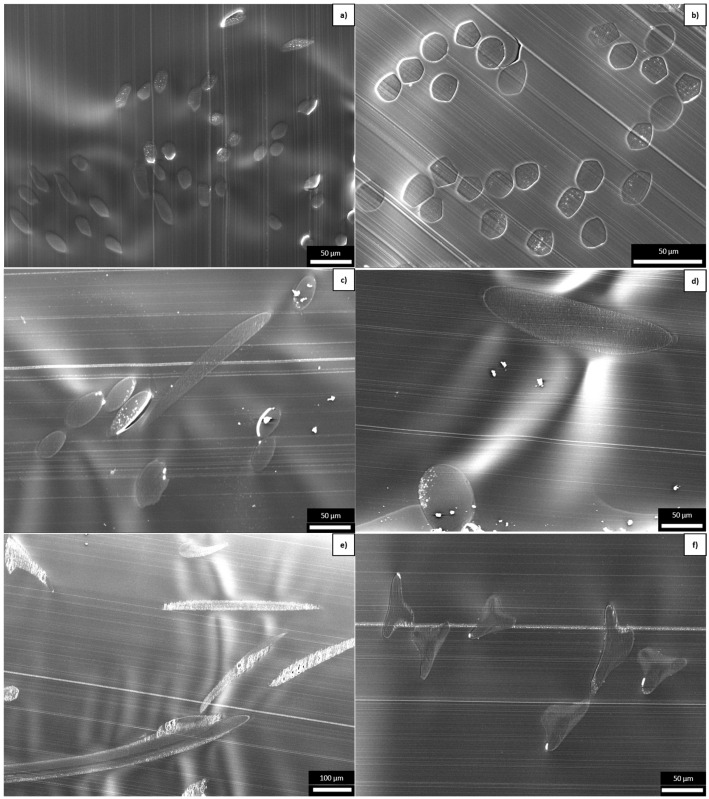
SEM images of cross-sections of the PLA fibers: (**a**) PLA_R1, (**b**) PLA_R2, (**c**) PLA_R3, (**d**) PLA_R4, (**e**) PLA_C, and (**f**) PLA_T.

**Figure 5:**
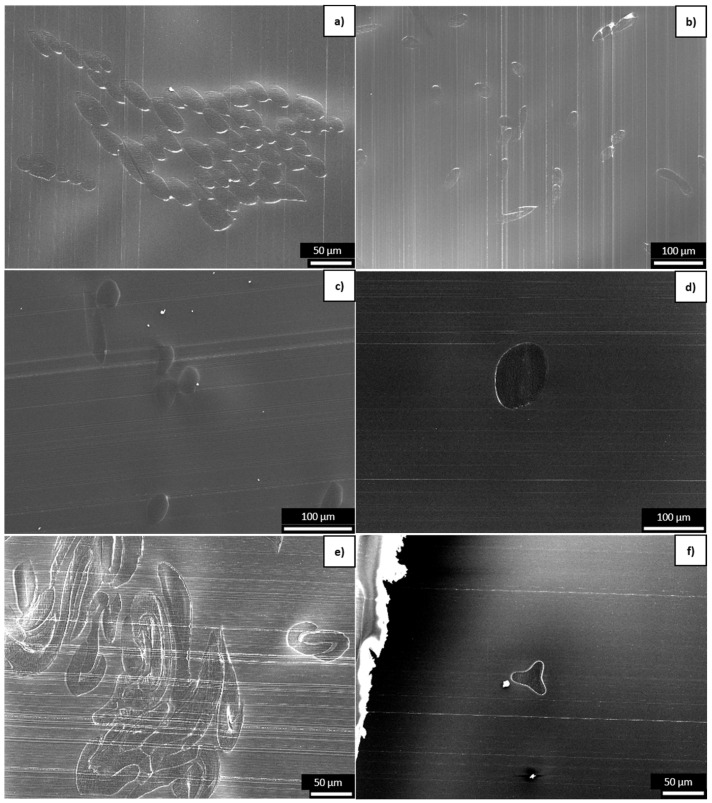
SEM images of cross-sections of the PBS fibers: (**a**) PBS_R1, (**b**) PBS_R2, (**c**) PBS_R3, (**d**) PBS_R4, (**e**) PBS_C, and (**f**) PBS_T.

**Figure 6:**
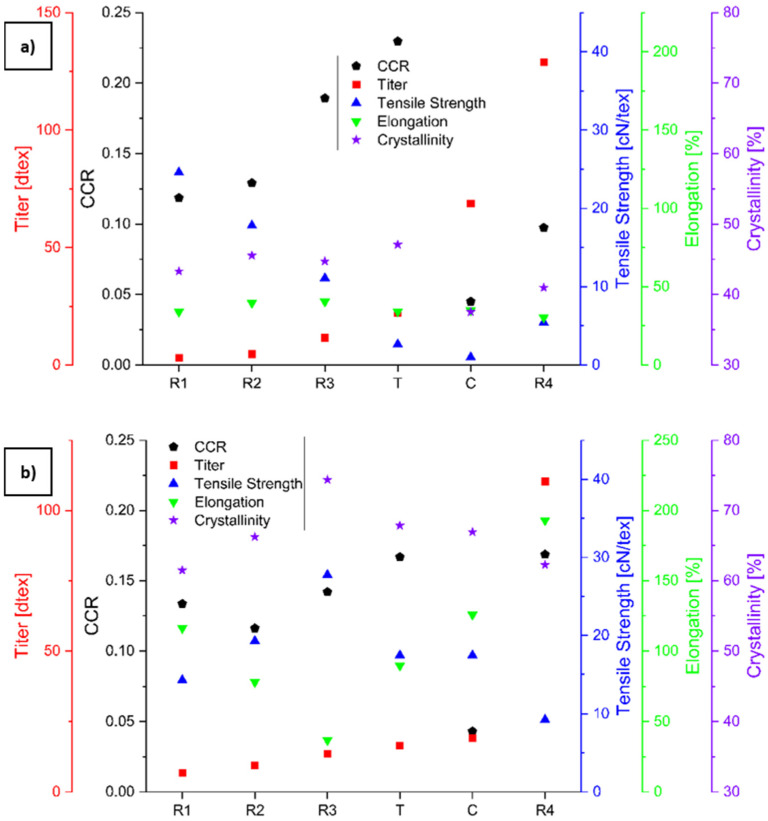
Fiber properties for (**a**) PLA and (**b**) PBS. The graphs use different symbols and scales to represent the CCR, titer, tensile strength, elongation, and crystallinity as shown in the key.

SEM images of the six different PLA fibers are shown in Figure 4 and equivalent images of the six different PBS fibers are shown in Figure 5. The CCR, titer, tensile strength, elongation, and crystallinity are shown in Figure 6 and are listed, along with the corresponding process parameters, in Table 2. The samples with circular cross-sections are listed by increasing circumference (thus decreasing CCR) but the more complex boundaries of the C-shaped and trilobal samples result in the following order: R1, R2, R3, T, C, and R4. The elongation at break was 30–40% for all PLA samples but much higher for the PBS samples, ranging from 36.84% for PBS_R2 to 193.05% for PBS_R4. PLA_R1 showed the highest tenacity (24.59 cN/tex), followed by PLA_R2 (17.81 cN/tex) and PLA_R3 (11.09 cN/tex). The most tenacious PBS sample was PBS_R3 (27.74 cN/tex). The least tenacious samples for each polymer were PLA_C (0.98 cN/tex) and PBS_R4 (9.24 cN/tex).

### 3.2. Changes Caused by Degradation

#### 3.2.1. Surface Changes Observed by SEM

**Figure 7:**
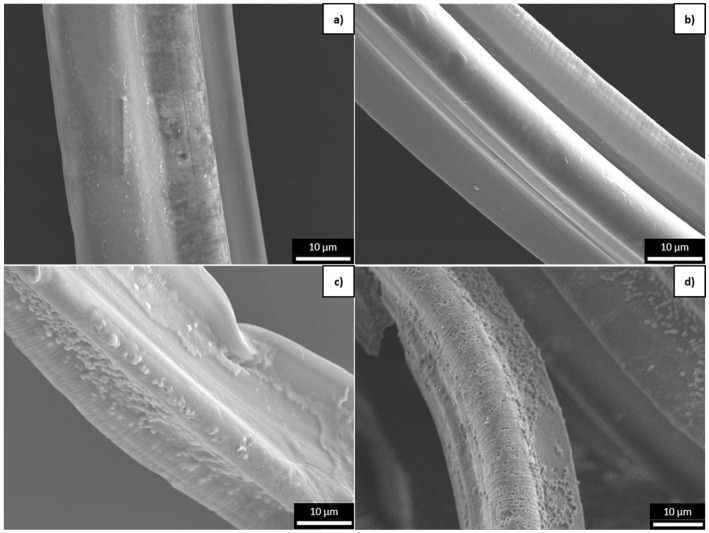
SEM images of the surface of PLA trilobal fibers at 2000× magnification. (**a**) Virgin PLA_T fiber before degradation. (**b**) PLA_T_300h fiber exposed to accelerated weathering. (**c**) Virgin PLA_T_ISO fiber subjected to disintegration without accelerated weathering. (**d**) PLA_T_300h+ISO fiber subjected to disintegration following exposure to accelerated weathering.

**Figure 8:**
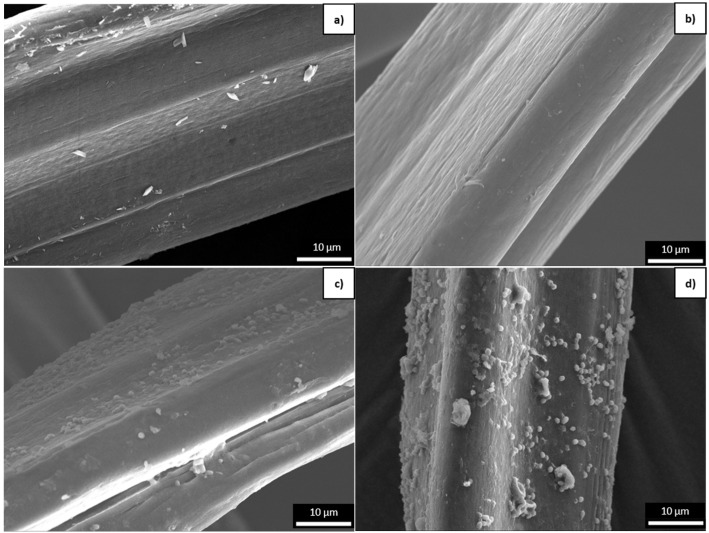
SEM images of the surface of PBS trilobal fibers at 2000× magnification. (**a**) Virgin PBS_T fiber before degradation. (**b**) PBS_T_300h fiber exposed to accelerated weathering. (**c**) Virgin PBS_T_ISO fiber subjected to disintegration without accelerated weathering. (**d**) PBS_T_300h+ISO fiber subjected to disintegration following exposure to accelerated weathering.

Surface changes during degradation were followed by SEM imaging of the trilobal fibers. The surface of the PLA_T fiber was initially smooth (Figure 7a) but small holes became apparent during accelerated weathering (Figure 7b). The absence of such holes in the virgin fibers slowed down the disintegration process (Figure 7c), whereas their presence was exploited during the disintegration of aged fibers, making the holes much larger (Figure 7d). Such openings were not observed in the PBS samples. However, the initially smooth surface (Figure 8a) became rougher during accelerated weathering (Figure 8b). The disintegration process also caused roughening of the surface (Figure 8c), which was exacerbated when the sample had been pre-aged (Figure 8d).

**Figure 9:**
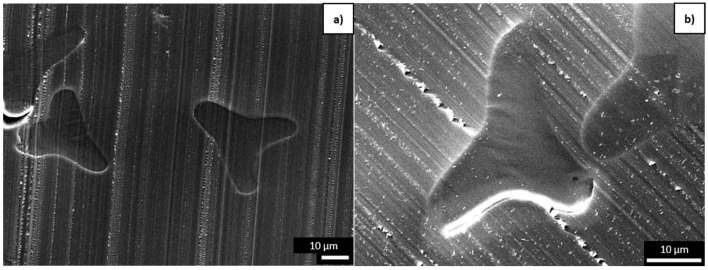

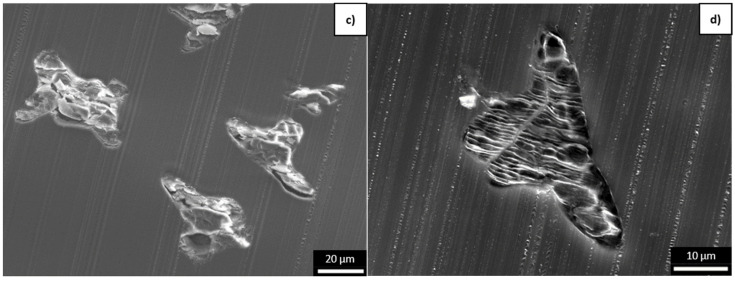
SEM images of PLA trilobal fiber cross-sections at 2000× magnification. (**a**) Virgin PLA_T fiber before degradation. (**b**) PLA_T_300h fiber exposed to accelerated weathering. (**c**) Virgin PLA_T_ISO fiber subjected to disintegration without accelerated weathering. (**d**) PLA_T_300h+ISO fiber subjected to disintegration following exposure to accelerated weathering.

When considering the cross-sections of the fibers, the PLA_T fibers appeared to become more brittle during degradation with or without aging (Figure 9c,d), whereas no overt change was observed for the PBS_T fibers.

#### 3.2.2. Changes in Thermal Properties Determined by DSC

**Figure 10:**
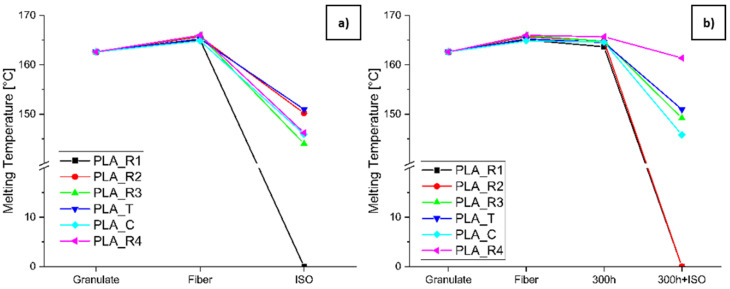
T_m_ change during the degradation of PLA along (**a**) degradation path 2.1 (granulate to fiber to ISO) and (**b**) degradation path 2.2 (granulate to fiber to 300h to 300h+ISO).

Sample degradation was accompanied by changes in the melting enthalpy peak, as shown by the measurement of T_m_, which was substantial for PLA (Figure 10) but only marginal for PBS. The T_m_ of PLA increased during the spinning process, and then dropped sharply as the sample was degraded. The T_m_ of PLA_R2 dropped below 145 °C after disintegration and thus represents the lowest T_m_ recorded for all samples. PLA_R1 degraded completely during disintegration testing, regardless of whether accelerated aging was applied or not, as did PLA_R2_300h+ISO.

**Figure 11:**
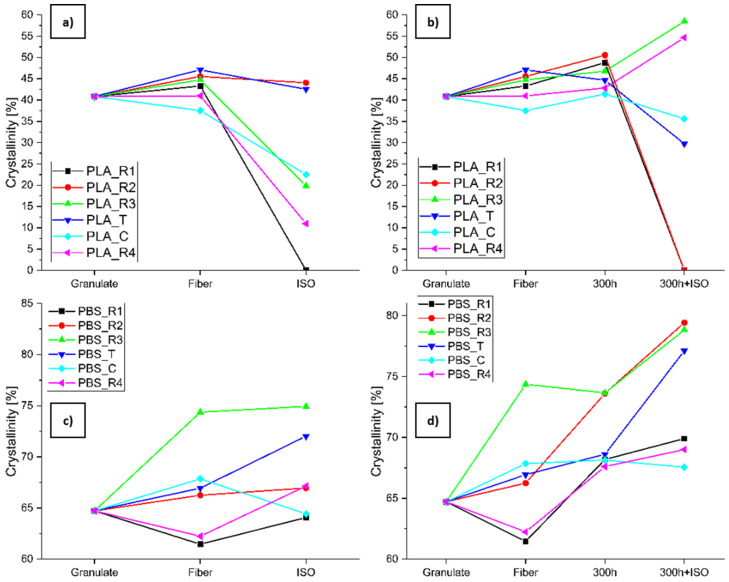
Change in crystallinity of (**a**,**b**) PLA and (**c**,**d**) PBS along (**a**,**c**) degradation path 2.1 (granulate to fiber to ISO) and (**b**,**d**) degradation path 2.2 (granulate to fiber to 300h to 300h+ISO).

The crystallinity of both polymers also changed during degradation (Figure 11). The crystallinity of all PLA samples except PLA_C increased during spinning (Figure 11a,b). In the absence of pre-aging, the crystallinity of all samples then dropped sharply during degradation (Figure 11a). In contrast, the crystallinity of most PLA samples increased slightly during accelerated weathering (only PLA_C decreased), and then most samples showed a sharp drop in crystallinity during degradation, although the crystallinity of PLA_R3 and PLA_R4 increased and reached the highest values recorded (Figure 11b). For PBS, the crystallinity of most samples increased during spinning, but decreased for PBS_R1 and PBS_R4 (Figure 11c,d). In the absence of pre-aging, all samples except PBS_C increased in crystallinity during degradation (Figure 11c). Accelerated aging caused the crystallinity of all samples except PBS_R3 to increase, followed by a further increase during degradation for all samples except PBS_C (Figure 11d). The crystallinity values are summarized in Table 3.

#### 3.2.3. Chemical Changes Determined by FTIR Spectroscopy

Changes in the hydroxyl, carbonyl, and vinyl groups of the polymers were determined by FTIR spectroscopy and the corresponding indices (CI, HI, and VI) are shown in Table 4, Table 5 and Table 6. The CI of all PLA samples was lower than for the corresponding PBS samples (Table 4). Furthermore, the CI of all PLA samples except PLA_R1 increased significantly from granule to fiber. The highest CI value of 1.85 was recorded for PBS_R1_300h. Interestingly, the CI values of PLA fibers generally increased in degradation pathway 2.1 (with only PLA_C decreasing), whereas the values generally decreased for PBS (with only PBS_R1 increasing). For pathway 2.2, there was no clear pattern for PLA fibers, with the CI increasing for two samples, decreasing for two others, and remaining undetermined for the final two samples because they were completely disintegrated. For PBS fibers, the CI generally decreased in pathway 2.2, although sample PBS_R1 showed the opposite behavior. The HI values (Table 5) of all PLA samples increased from granule to fiber, with sample PLA_R1 showing a small change and the HI doubling for all other samples. PLA_R2 fibers scored the highest value for any of the indices in the entire study (HI = 2.01). However, no major changes were observed when the PBS granules were spun into fibers. Similarly, there was no noticeable change in HI during the degradation of any of the fibers. The VI values (Table 6) of most PLA samples increased from granule to fiber, with sample PLA_R1 exceptionally showing a small decrease, but there was no further change during degradation. For PBS, the VI values did not change notably during fiber spinning or degradation.

#### 3.2.4. Changes in M_n_ Determined by GPC

**Figure 12:**
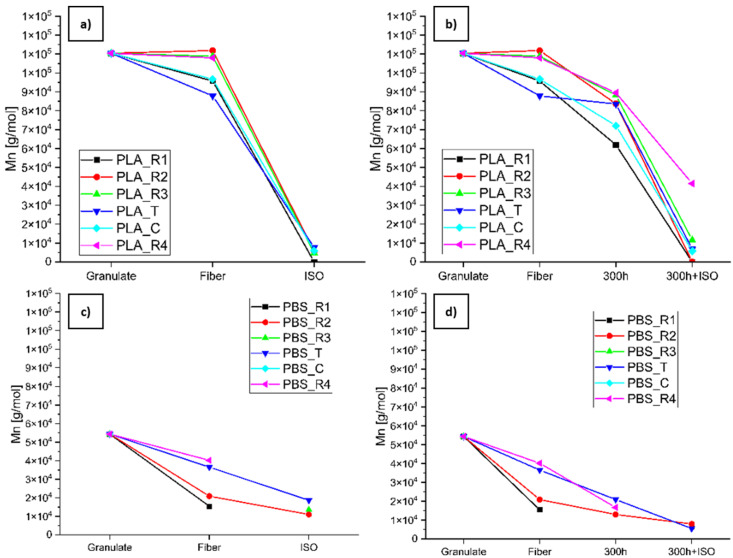
Changes in the M_n_ of (**a**) PLA and (**b**) PBS along (**a**,**c**) degradation path 2.1 (granulate to fiber to ISO) and (**b**,**d**) degradation path 2.2 (granulate to fiber to 300h to 300h+ISO).

The PLA samples were dissolved in chloroform, whereas the PBS samples were dissolved in hexafluoro-2-isopropanol (HFIP) where possible. Several PBS samples (PBS_R1_300h, PBS_R3, PBS_R3_300h, PBS_R3_300h+ISO, PBS_C, PBS_C_300h, PBS_C_300h+ISO, PBS_R1_ISO, PBS_R4_ISO, and PBS_C_ISO) were insoluble in HFIP as well as chloroform, tetrahydrofuran, and dimethylformamide. A substantial decrease in M_n_ was evident for most samples along both degradation paths (Figure 12). PLA_R2 was the only sample that showed a marginal increase in M_n_ during the spinning process (Figure 12a,b). PLA_R4_300h, PLA_R3_300h, and PLA_R2_300h showed the lowest impact of aging of all PLA samples, whereas PLA_R1 showed the steepest decline in M_n_ due to accelerated weathering (Figure 12b). The M_n_ of all the PBS samples declined during spinning (Figure 12c,d) and showed significantly greater UV sensitivity than the PLA samples (Figure 12d). The M_n_ of the aged PBS samples declined more than that of the virgin samples during disintegration (Figure 12c,d).

The degradation parameter *K* was calculated for degradation paths 2.1 (Table 7) and 2.2 (Table 8) using polymer granules as the comparator to provide an overview of PLA and PBS degradation. Changes during the spinning process are well known for PLA [36] but are less apparent for PBS. When degradation was also considered, UV exposure had a major effect on the PBS samples, whereas disintegration and combined UV pre-loading and disintegration both strongly affected the PLA samples. For degradation path 2.1 (Table 7), the highest *K* values for each polymer as a fiber (not including fully degraded samples) were 1.257 for PLA_T and 3.515 for PBS_R1, whereas the highest values after degradation were 23.179 for PLA_R3 and 4.927 for PBS_R2. For degradation path 2.2 (Table 8), the PBS samples had higher *K* values than the PLA samples after accelerated weathering, suggesting they were more sensitive to UV photolysis and hydrolysis. However, the highest calculated *K* values after aging and disintegration (not including fully degraded samples) were 19.04 for PLA_C and 9.752 for PBS_T, showing that the impact of accelerated aging is higher for PLA than PBS. PLA_R1 and PLA_R2_300h+ISO were completely disintegrated, confirming the greater sensitivity of PLA.

#### 3.2.5. Impact of Accelerated Weathering in Connection with the CCR

**Figure 13:**
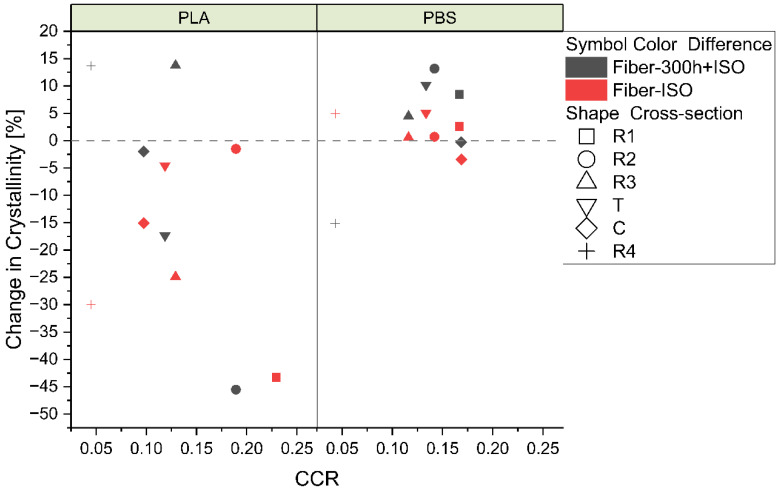
Comparative change in the crystallinity of PLA and PBS polymers in degradation path 2.1 (red) and degradation path 2.2 (gray).

The change in polymer crystallinity as a function of the CCR differed considerably between PLA and PBS for both degradation paths (Figure 13). PLA showed a steeper decrease in crystallinity with an increasing CCR, whereas this correlation was not observed for PBS. All PBS samples except PBS_R4_300h+ISO and PBS_C_ISO increased in crystallinity, whereas all PLA samples except PLA_R4_300h+ISO and PLA_R3_300h+ISO decreased in crystallinity. PLA_R1 in both degradation paths, as well as PLA_R2_300h+ISO, showed the greatest change in crystallinity because the samples disintegrated completely.

**Figure 14:**
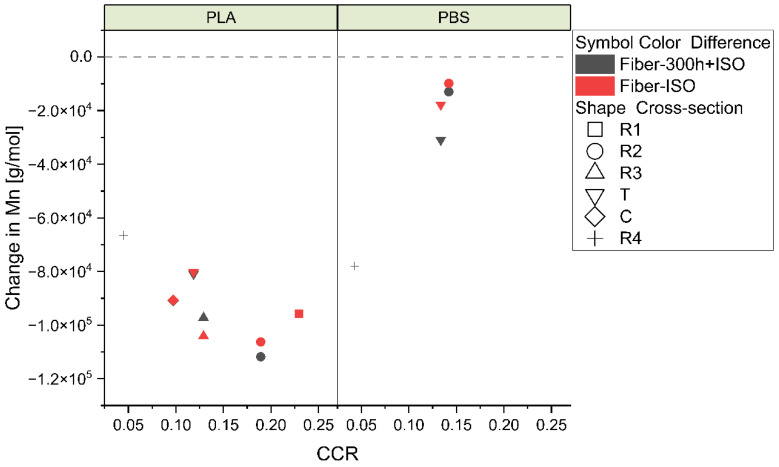
Comparative change in the M_n_ of PLA and PBS polymers in degradation path 2.1 (red) and degradation path 2.2 (gray).

PLA also showed a more considerable decrease in M_n_ overall compared to PBS. In both degradation paths, a larger CCR resulted in a more noticeable change in M_n_ for PLA, whereas PBS showed the opposite behavior, where a greater decrease in M_n_ was observed for lower CCR values (as shown in Figure 14).

## 4. Discussion

### 4.1. Fiber Properties

We compared a range of fibers differing in their circumference and cross-sectional shape (represented by the CCR) to determine the impact of these parameters on fiber degradation. A larger CCR equates to a larger surface area and a lower cross-sectional area, which leads to more efficient degradation (Figure 13). The cross-section of R1 fibers corresponded to the highest CCR (0.167 for PBS_R1 and 0.22961 for PLA_R1), whereas that of the R4 fibers corresponded to the lowest (0.04305 for PBS_R4 and 0.04504 for PLA_R4). The circumference of each fiber was used to calculate the titer and to compare the tensile strength (cN/tex). PBS_R3 formed the strongest fibers (27.74 cN/tex), followed by PLA_R1 (24.59 cN/tex) and PLA_R2 (17.81 cN/tex) (Figure 6). The elongation at break tended to be lower for PLA than PBS fibers with a matching cross-section. Furthermore, PLA fibers tended to increase in tenacity with a decreasing CCR, whereas PBS fibers tended to increase in tenacity until a certain CCR (in this case PBS_R3) and then decrease again. PBS formed more crystalline fibers, with PBS_R3 showing the highest crystallinity (74.38%). PLA_T was the most crystalline of the PLA fibers (47.08%). The physical performance of the fibers (higher tensile strength, lower elongation at break, and higher crystallinity) was linked to a higher CCR. PLA appeared to be more process sensitive than PBS based on the CI, HI, and VI (calculated from FTIR data) as well as the change in M_n_.

### 4.2. Change Due to Degradation

We observed a massive change in T_m_ and M_n_ during the degradation of all PLA samples, in agreement with earlier reports [48,49]. The T_m_ change was lower for the PBS samples. Furthermore, the degradation of PLA induced by the spinning process was evident in the calculated CI, HI, and VI. The CI and VI of PLA increased during spinning and decreased slightly during degradation [50]. The expected increase in crystallinity was observed for samples PBS_R4, PLA_C, PBS_C, PLA_R3, PBS_R3, and PBS_R2 in degradation pathway 2.1. As well as the degradation of amorphous structures, further crystallization of the PBS samples is possible when the glass transition temperature (T_g_) is exceeded [51]. The same phenomenon was observed for degradation path 2.2 in the case of PLA_T and PBS_T. An increase in crystallization after accelerated weathering was observed for all samples except PLA_R2, PLA_C, PBS_C, and PLA_R4.

The PLA materials with the highest and second highest CCR degraded completely when exposed to accelerated weathering, and PLA showed a greater change in crystallinity due to degradation than PBS. With the exception of PLA_R3 and PLA_R4 in degradation path 2.1, all PLA samples decreased in crystallinity during degradation, which has also been observed in other studies [48]. The M_n_ followed a similar decreasing trend during degradation. In contrast, the crystallinity of most PBS samples increased during degradation, the exception being PBS_C, indicating that the amorphous regions in the polymer were degraded first. The change in M_n_ for PBS samples was also lower than for PLA, with the exception of PBS_R4. The decrease in crystallinity for some of the PLA samples during degradation may reflect a change in the L/D ratio, given that the L structure is more amorphous, and could influence subsequent degradation behavior [52,53,54].

The CCR had a more notable impact on the properties of PLA than PBS during degradation. PLA shows a tendency for a greater decrease in crystallinity and M_n_ with an increasing CCR regardless of the degradation path. This is in line with expectations because a more crystalline content is more difficult to break down. The smaller change in the PBS samples may reflect the already lower M_n_ in the PBS fibers before degradation. The degradation temperature exceeded the T_g_ of the PBS samples, allowing further crystallization [55], and could thus influence the overall degradation behavior.

## 5. Conclusions

The results of this study are summarized in Table 9 for PLA and Table 10 for PBS. Previous degradation studies have shown that the CI and HI increase [56,57], as does the crystallinity, whereas the T_m_ and M_n_ decrease [58,59,60]. For the PLA samples, we identified a correlation between a higher CCR and more efficient degradation, whereas the PBS samples showed the opposite relationship. More significant degradation was observed for PLA than PBS when exposed to accelerated weathering in degradation path 2.2. A larger CCR corresponds to a larger fiber surface area compared to the cross-sectional area, and therefore accelerates degradation by increasing the accessibility of the polymer [15]. Degradation increases the crystallinity of PBS, perhaps because the amorphous regions are degraded first or because the test conditions exceeded the T_g_, which would induce fresh crystallization. In contrast, degradation reduces the crystallinity of PLA, indicating that the amorphous and crystalline regions are broken down at the same time. Other studies [7,53] have shown that PBS samples are more crystalline than PLA. Three of the PLA samples were completely disintegrated at 38 °C in 45 days, showing that PLA is more susceptible to degradation under environmental conditions than PBS. Given the correlation between degradation and T_g_, PLA degradation is anticipated to commence at 60 °C [34].

Hydrolysis and UV exposure had a notable impact on the samples prior to degradation, consistent with previous studies [61]. Disintegration begins on the surface and progresses inwards, and is expedited by surface changes induced by UV exposure [62]. The UV-pre-exposed samples showed overt surface changes after the disintegration test, as revealed by SEM images. Accelerated weathering in the form of UV photolysis and hydrolysis open the surface and ultimately enhance the degradation of the sample. Notably, PLA_R1 and PLA_R2 have a large surface area prone to accelerated weathering. Previous degradation tests have been applied to virgin samples, which only partially depict real-world behavior.

Beyond the CCR, various factors such as textile structure, temperature, and humidity contribute to degradation. This study normalized the CCR to determine its influence on degradation. Our findings suggest that degradability could be modulated by controlling the relationship between fiber surface and cross-sectional area, presenting opportunities to transform biopolymer production methods, particularly in single-use products such as melt-binding fibers.

## Figures and Tables

**Table 1:** Properties of fiber samples.

Sample	Extrusion Temperature [°C]	Extruder Speed [/min]	Melt Draw Ratio	Draw Ratio	Overall Draw Ratio	Throughput [g/min]
PLA_R1	245	20	0.59	2.4	2.99	11.66
PLA_R2	240	20	0.44	2.9	3.34	11.66
PLA_R3	245	25	0.95	2.3	3.25	14.575
PLA_R4	245	20	0.69	2.9	3.59	11.66
PLA_C	240	20	0.44	2.9	3.34	11.66
PLA_T	240	20	1.50	1.9	3.40	11.66
PBS_R1	215	20	0.67	3.2	3.87	11.66
PBS_R2	215	20	2.61	2.9	5.51	11.66
PBS_R3	215	20	1.33	3.2	4.53	11.66
PBS_R4	217	20	0.82	3.1	3.92	11.66
PBS_C	205	20	0.79	2.9	3.69	11.66
PBS_T	215	20	1.02	2.9	3.92	11.66

**Table 2:** Machine settings and fiber physical properties. Samples are grouped by cross-section and are shown in order of increasing circumference, with gray shading highlighting the PLA samples.

Sample	Extrusion Temperature [°C]	Overall Draw Ratio	Circumference [µm]	Titer [dtex]	CCR	Tensile Strength [cN/tex]	Elongation [%]	Crystallinity [%]
PLA_R1	245	2.99	52.78	2.73	0.229	24.59	34.01	43.29
PBS_R1	215	3.87	58.27	3.33	0.167	14.28	116.21	61.46
PLA_R2	240	3.34	65.21	4.17	0.189	17.81	39.63	45.53
PBS_R2	205	3.69	87.94	7.58	0.142	19.28	78.05	66.25
PLA_R3	245	3.25	110.79	12.04	0.129	11.09	40.48	44.71
PBS_R3	215	4.53	121.65	14.51	0.116	27.74	36.84	74.38
PLA_T	240	3.4	187.85	34.6	0.119	2.64	34.1	47.08
PBS_T	215	3.92	154.86	23.51	0.134	17.44	89.59	67.85
PLA_C	240	9.59	276.67	75.06	0.097	0.98	34.82	37.53
PBS_C	215	5.51	248.02	60.32	0.169	17.45	125.76	66.94
PLA_R4	245	3.59	301.74	89.28	0.045	5.42	30.34	40.96
PBS_R4	217	3.92	327.29	105.04	0.043	9.24	193.05	62.24

**Table 3:** Changes in crystallinity due to degradation along path 2.1 (orange) and 2.2 (blue). N.A. indicates fully degraded samples where crystallinity could not be measured.

	Path	PLA_R1	PLA_R2	PLA_R3	PLA_T	PLA_C	PLA_R4	PBS_R1	PBS_R2	PBS_R3	PBS_T	PBS_C	PBS_R4
Granulate		40.82	40.82	40.82	40.82	40.82	40.82	64.72	64.72	64.72	64.72	64.72	64.72
Fiber		43.29	45.53	44.71	47.08	37.53	40.96	61.46	66.25	74.38	66.94	67.85	62.24
ISO	2.1	N.A.	44.04	19.81	42.53	22.49	10.97	64.06	66.96	74.94	72.03	64.43	67.14
300h	2.2	48.78	50.53	46.79	44.66	41.4	42.82	69.90	73.61	73.66	68.62	68.15	67.61
300h+ISO	2.2	N.A.	N.A.	58.45	29.73	35.58	54.65	64.72	79.42	78.82	77.11	67.55	68.99

**Table 4:** Carbonyl indices (CIs) of PLA and PBS at different stages of fiber production and degradation via pathways 2.1 (orange) and 2.2 (blue).

Sample	Path	PLA R1	PLA R2	PLA R3	PLA T	PLA C	PLA R4	PBS R1	PBS R2	PBS R3	PBS T	PBS C	PBS R4
Granulate		0.51	0.51	0.51	0.51	0.51	0.51	1.33	1.33	1.33	1.33	1.33	1.33
Fiber		0.53	1.06	1.05	1.05	1.03	1.02	1.62	1.61	1.63	1.69	1.77	1.74
ISO	2.1	N.A.	1.10	1.11	1.06	1.01	1.06	1.65	1.53	1.52	1.63	1.75	1.49
300h	2.2	0.56	1.09	1.07	1.07	1.07	1.05	1.85	1.58	1.57	1.62	1.65	1.75
300h+ISO	2.2	N.A.	1.03	1.08	1.04	N.A.	1.12	1.70	1.49	1.59	1.44	1.51	1.66

**Table 5:** Hydroxyl indices (HIs) of PLA and PBS at different stages of fiber production and degradation via pathways 2.1 (orange) and 2.2 (blue).

Sample	Path	PLA R1	PLA R2	PLA R3	PLA T	PLA C	PLA R4	PBS R1	PBS R2	PBS R3	PBS T	PBS C	PBS R4
Granulate		0.50	0.50	0.50	0.50	0.50	0.50	0.93	0.93	0.93	0.93	0.93	0.93
Fiber		0.48	2.01	0.97	0.96	0.95	0.97	0.92	0.92	0.92	0.92	0.91	0.92
ISO	2.1	N.A.	0.98	0.98	0.98	0.97	0.98	0.94	0.94	0.94	0.93	0.92	0.95
300h	2.2	0.48	0.98	0.98	0.97	0.96	0.96	0.91	0.93	0.92	0.93	0.92	0.91
300h+ISO	2.2	N.A.	0.98	0.98	0.98	N.A.	0.95	0.94	0.95	0.93	0.96	0.95	0.93

**Table 6:** Vinyl indices (VIs) of PLA and PBS at different stages of fiber production and degradation via pathways 2.1 (orange) and 2.2 (blue).

Sample	Path	PLA R1	PLA R2	PLA R3	PLA T	PLA C	PLA R4	PBS R1	PBS R2	PBS R3	PBS T	PBS C	PBS R4
Granulate		0.51	0.51	0.51	0.51	0.51	0.51	1.15	1.15	1.15	1.15	1.15	1.15
Fiber		0.50	1.01	0.97	0.99	0.96	0.97	1.13	1.16	1.13	1.16	1.18	1.15
ISO	2.1	N.A.	1.02	1.00	1.00	0.97	1.01	1.15	1.15	1.17	1.18	1.19	1.15
300h	2.2	0.50	1.01	0.99	1.01	1.00	0.98	1.18	1.18	1.15	1.17	1.17	1.20
300h+ISO	2.2	N.A.	0.99	1.00	0.98	N.A.	1.04	1.18	1.16	1.17	1.16	1.17	1.20

**Table 7:** Degradation parameter *K* calculated for degradation path 2.1. D = fully degraded. N.A. = insoluble so no results available.

Sample	Fiber	ISO
PLA_R1	1.153	D
PLA_R2	0.987	19.84
PLA_R3	1.014	23.179
PLA_T	1.257	11.535
PLA_C	1.142	18.454
PLA_R4	1.023	N.A.
PBS_R1	3.515	N.A.
PBS_R2	2.601	4.927
PBS_R3	N.A.	4.022
PBS_T	1.488	2.905
PBS_C	N.A.	N.A.
PBS_R4	1.332	N.A.

**Table 8:** Degradation parameter *K* calculated for degradation path 2.2. D = fully degraded. N.A. = insoluble so no results available.

Sample	Fiber	300h	300h+ISO
PLA_R1	1.153	1.781	D
PLA_R2	0.987	1.319	D
PLA_R3	1.014	1.251	9.505
PLA_T	1.257	1.321	16.036
PLA_C	1.142	1.529	19.04
PLA_R4	1.023	1.234	2.662
PBS_R1	3.515	N.A.	8.16
PBS_R2	2.601	4.179	6.849
PBS_R3	N.A.	N.A.	N.A.
PBS_T	1.488	2.596	9.752
PBS_C	N.A.	N.A.	N.A.
PBS_R4	1.332	3.253	N.A.

**Table 9:** Summary of results for PLA. Arrows in the *Change toward* column indicate the direction of change in the parameters listed on the left (↑ = significant increase, ↓ = significant decrease, ↗ = increase, - = constant, ↘ = decrease, and yes = applied). Arrows in the fiber property columns indicate the effect of these parameters. Gray cells indicate effects that were not tested. Green shading indicates process parameters that we controlled.

Parameter	Change Towards	Crystallinity	Tenacity	Elongation	CI	VI	HI	M_n_	Degradability
CCR	↑	-	↘	-	↘	↗	↗	↘	↑
Draw ratio	↗	↗	↗	↘	↘	↗	↘	-	↘
Tenacity	↑	↗		↘	↗	↗	↘	-	↘
Elongation	↘	↗	-		↘	↗	↗	↘	↗
UV exposure before disintegration	Yes	↗			↘	↓	↓	↓	↑

**Table 10:** Summary of results for PBS. Arrows in the *Change toward* column indicate the direction of change in the parameters listed on the left (↑ = significant increase, ↓ = significant decrease, ↗ = increase, - = constant, ↘ = decrease, and yes = applied). Arrows in the fiber property columns indicate the effect of these parameters. Gray cells indicate effects that were not tested. Green shading indicates process parameters that we controlled.

Parameter	Change Towards	Crystallinity	Tenacity	Elongation	CI	VI	HI	M_n_	Degradability
CCR	↑	↗	↘	↗	↘	↘	↗	↗	↘
Draw ratio	↗	-	-	-	↘	↗	↘	↓	↗
Tenacity	↑	↗		↘	↘	↘	↗	↘	↘
Elongation	↘	-	↗		↘	↘	↘	↓	↗
UV exposure before disintegration	Yes	↗			↘	↘	↗	↓	↘

## Data Availability

The original contributions presented in the study are included in the article, further inquiries can be directed to the corresponding author.
